# Ego and Spiritual Transcendence: Relevance to Psychological Resilience and the Role of Age

**DOI:** 10.1155/2013/949838

**Published:** 2013-10-09

**Authors:** Barbara Hanfstingl

**Affiliations:** School of Education, Alpen-Adria-Universität Klagenfurt, Sterneckstraße 15, 9020 Klagenfurt, Austria

## Abstract

The paper investigates different approaches of transcendence in the sense of spiritual experience as predictors for general psychological resilience. This issue is based on the theoretical assumption that resilience does play a role for physical health. Furthermore, there is a lack of empirical evidence about the extent to which spirituality does play a role for resilience. As potential predictors for resilience, ego transcendence, spiritual transcendence, and meaning in life were measured in a sample of 265 people. The main result of a multiple regression analysis is that, in the subsample with people below 29 years, only one rather secular scale that is associated with ego transcendence predicts resilience, whereas for the older subsample of 29 years and above, spiritual transcendence gains both a positive (oneness and timelessness) and a negative (spiritual insight) relevance to psychological resilience. On the one hand, these results concur with previous studies that also found age-related differences. On the other hand, it is surprising that the MOS spiritual insight predicts psychological resilience negatively, the effect is increasing with age. One possible explanation concerns wisdom research. Here, an adaptive way of dealing with the age-related loss of control is assumed to be relevant to successful aging.

## 1. Introduction

In an overview, Seybold and Hill [1] point out that, first, there are many different dimensions which are attributed to religiousness or spirituality. Second, there are both helpful and harmful effects of spirituality, but the influence is largely beneficial. In fact, this is a very general statement about the influence of spirituality. Third, psychological factors such as coping strategies or cognitive processes (e.g., locus of control) may mediate the relationship between spirituality and health. Seybold and Hill explicitly call for an investigation of the role of psychological factors as possible mediators in the religiousness-health connection. On a theoretical basis, psychological resilience is assumed to be a possible mediator between spirituality and health. In the present study the empirical focus lies on the identification of spiritual dimensions as predictors of resilience. Spiritual dimensions here represent especially transcendental experiences.

Since Werner [2] first came into contact with children expressing high psychological resilience, the construct has become an important research area in positive psychology. While earlier theories focused on innate facets of resilience, current approaches concentrate on learnable and environmental factors. Recently, the acquisition of higher resilience has become an aspired goal in psychotherapy (e.g., [3]). It is not yet sufficiently clear to what extent spirituality and transcendental experiences enhance or foster psychological resilience. Thus, the first purpose of this study is to analyze which aspects of transcendence could have a positive or negative influence on general resilience. Different aspects of transcendence, in this study, are approaches with different traditions and understandings of transcendence or spirituality in psychological research. The first approach, related to personality psychology, focuses on the quality of mental information processing and basics of motivational forces [4]. In this paper, this type of transcendence is called ego transcendence [5]. In contrast, the second approach follows more traditional spirituality research that emanates from mysticism research [6–8]. This approach here is labeled spiritual transcendence. A more phenomenological and modern approximation to spirituality comes from Schnell [9], on the basis of research on contemporary resources of meaning in life.

Up to now, differences between different conceptualizations of spirituality or transcendence in predictive power concerning resilience have not yet been addressed. So, the first main purpose of this study is to explore these different relevancies of spiritual approaches. The second aim is to identify age-related differences in the predictive power of the different approaches. This second research question is important because Büssing and his colleagues [10] reported a meaningful age difference: whereas for older people transcendental beliefs played an important role in life, adolescents tended to focus more on more secular value orientations (see also [11]). In the following, psychological resilience and the three approaches to spirituality or transcendence are described in detail.

### 1.1. Resilience

Basically, resilience is a broad construct that has no single definition and subsumes different aspects of psychological resistance. Werner and Smith [2] entitled their book Vulnerability Without Invincibility, and wrote about children that were capable of living a successful life in spite of adverse and difficult circumstances. In an overview article, Richardson [12] identified three waves of resilience research. The first wave was characterized through the phenomenological clarification of developmental domains and protective factors. The second wave concentrated on disruptive and reintegrative processes for acquiring resilient qualities, whereas the third wave emphasized a postmodern and multidisciplinary view on resilience. A state-of-the-art view on resilience was presented by Fröhlich-Gildhoff and Rönnau-Böse [13, page 13]. They defined resilience as a “dynamic or compensatory process of positive adaptiveness in the face of inauspicious development and the emergence of load factors.” Here, inauspicious development means an ontogenetic development in the face of a high number of psychological risk factors. “Load factor” means the pressure and strain caused by these risk factors. Furthermore, the authors emphasized that resilience is a variable dimension including multidimensional situational factors. Wagnild and Young [14] described resilience as a personality factor that moderates negative emotions and distress and facilitates a flexible adaption to suboptimal conditions.

There is abundant empirical evidence that psychological resilience helps to regain or maintain physical health. For example, Yi et al. [15] demonstrated that people with high resilience scores did not show any association between rising distress and worsening glycosylated hemoglobin, whereas the group with low or moderate resilience showed a strong association. Nygren et al. [16] found that resilience had significant positive correlations with the sense of coherence and self-transcendence as well as with perceived physical and mental health in a sample of very old (85+) people. Leppert et al. [17] identified resilience as a protective personality factor in old people. Taking a look into cancer and palliative research, Strauss et al. [18] showed that higher resilience was accompanied by positive stress management during radiation therapy. Another example of the relevance of psychological resilience was presented by Tugade et al. [19], based on the assumption that resilience is accompanied by positive emotions [20]. They demonstrated that psychologically resilient people rebounded from negative emotional arousal through their experience of positive emotions. Furthermore, their findings showed a clear positive correlation between trait resilience and faster cardiovascular recovery, mediated by positive emotions. The current work aims to concretize the connection between different facets of transcendence and trait resilience, depending on age. In the following, the theoretical rationale of the difference between ego and spiritual transcendence, based on the theoretical assumptions of Kuhl and Fuhrmann [4], is outlined.

Kuhl and Fuhrmann [4, 21] postulated that human personality can be seen as a conglomeration of inner processes of regulative systems. The core of his personality interaction theory (PSI theory) is the assumption that personality comprises two modes of information processing: the “explicit self-regulation system” and the “implicit self-regulation system.” These two subsystems have different functions with regard to the mental regulation of a person. The *explicit self-regulation system* is called “ego” and focuses on maintaining the individual's intended goals. It is directed towards the future and comprises the conscious, analyzing part of the mind as well as the so-called “intention memory.” Following PSI theory, explicit self-regulation is associated with negative or decreasing positive affect. In contrast to this, the *implicit self-regulation system* according to Kuhl is labeled as “self” and has the function to maintain the self of a person. The “extension memory”—also called implicit memory—is supposed to be an executing part of it. It is responsible for the holistic feeling of the self and comprises memories and “cognitive maps” that represent a person's self-congruent autobiographical content [21, 22]. According to PSI theory, the implicit self-regulation system is activated by positive or decreasing negative affect. As theoretically expected, implicit but not explicit self-regulation plays an important role for job-related intrinsic motivation [23].

### 1.2. Implicit Self-Regulation as Ego Transcendence

**Figure 1:**
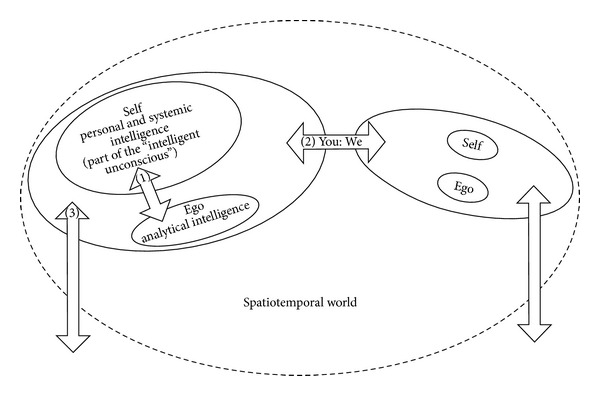
Three kinds of transcendence. (1) Ego transcendence (self: beyond ego), (2) self-transcendence (beyond the self: the other), and (3) spiritual transcendence (beyond space and time). Adapted version based on Kuhl [5, page 23].

Kuhl distinguished ego, self-, and spiritual transcendence [5].  Figure 1 shows that ego transcendence means the capability to transcend and overcome the barrier between the two functional systems called implicit and explicit self-regulation.

Self-transcendence is assumed to be the transcendence between I and You, in the sense of the capability to get involved with another person, whereas spiritual transcendence refers to the transcendence into a world beyond the spatiotemporal world that surrounds us.

Implicit self-regulation is characterized by parallel and holistic processing of complex conscious and nonconscious self-related information. People with well-developed implicit self-regulative competencies have access to this information, not in a common analytical but more in a feeling-oriented intuitive way. Being in an implicit regulative mode means handling these issues in a way that allows the self to be constructed congruently. According to Kuhl, overcoming the barrier between ego and self—therefore ego transcendence—is, for example, switching willfully between an analytical (intention memory) and a holistic (extension memory) information processing. Kuhl and colleagues [4, 24] postulate that implicit self-regulation is closely linked to spirituality. Thus, the people could subjectively experience ego transcendence as a kind of spirituality. In contrast to this, mystical experience is assumed to involve spiritual transcendence and a perception of divinity, as described in the following.

### 1.3. Mystical Orientation as Spiritual Transcendence

Francis and Louden [8, page 100] mentioned that there is no consistent definition in the literature of what “mysticism-in-general” is. They characterized mysticism “as a sense of union or identity with something other than oneself.” In this way, mysticism can be found in many different religious and spiritual systems. Francis and Louden identified mysticism as the core of all religions. On the basis of this definition, spiritual transcendence is well discriminable from ego transcendence as described above.

### 1.4. New Structure of MOS

In order to measure aspects of mysticism, Francis and Louden developed their Mystical Orientation Scale (MOS) based on Happold's seven aspects of mysticism [6]: ineffability, noesis, transiency, passivity, oneness, timelessness, and true ego. As an aside, the first four aspects were also mentioned by James [7] in regard to mysticism. Francis and Louden constructed three items for each facet, so the MOS comprises 21 items. The original MOS is conceptualized as a one-dimensional scale, and it has been validated on a sample of 3581 Catholic priests. However, for samples of laypeople, mysticism would be overdifferentiated if it is compartmentalized into seven aspects. This assumption is supported by a test-statistical analysis of the seven original MOS: only a few of the seven scales reached satisfying internal consistencies [25, page 22]. Schnell and Hanfstingl [26] translated the original items into German and had them translated back into English by a native speaker. In a further step, Hanfstingl and Römer [25] conducted a validation study with a sample of religious and spiritual laypeople. In a principal component analysis with varimax rotation, they identified three components of mysticism: oneness and timelessness, (perception of) good power, and spiritual insight. These factors are actually close to the original aspects of mysticism postulated by James and Happold. *Oneness and timelessness* factor reflects the two dimensions already described by Happold, but the factor also contains aspects of true ego. It describes a kind of mysticism that is characterized by feeling unified with all existing world and time, with the past and future. Thus, spirituality here means a feeling of being merged with the whole world and time. *Good power* emphasizes the feeling that one is positively influenced by a power outside of one's control, which may be reflected, for example, in aspects of passivity. Both the scales oneness and timelessness and good power reflect a kind of spirituality that involves a positively perceived tolerance of being controlled externally. *Spiritual insight* includes aspects of noesis as well as ineffability and transiency. In contrast to the other two MOS, it comprises items which emphasize retaining personal control in situations, feelings, or qualities that contain a deep truth or an insight in a higher plane. It characterizes a person who “stays human” in realizing spiritual experience, retaining control over the experience.

To conclude, it makes sense not to conceive of mysticism as unidimensional, although the internal consistency of the German MOS as a whole is very satisfactory with *α* = .93. Furthermore, the three scales oneness and timelessness, good power, and spiritual insight provide a good description of discriminable facets of mysticism that seem to have differential relevance to spiritual laypeople. Next, an additional aspect of spirituality and/or spiritual experience is described that is assumed to have relevance to resilience.

### 1.5. Meaning in Life

Schnell highlighted the importance of the meaning in life as a substantial component of well-being [9]. In line with Frankl [27], she emphasized the relevance of meaning in life for living a fulfilled life. Schnell and Becker [28] found that the most important predictor of meaningfulness is self-transcendence, but they did not define self-transcendence in the sense of Kuhl [5]. In the study by Schnell and Becker, vertical self-transcendence consisted of explicit religiosity and spirituality, whereas horizontal self-transcendence comprised unison with nature, social commitment, generativity, care for others, and health [29]. In the following, having meaning in life in the study was assumed to positively influence resilience, for which meaning in life and crisis of meaning were measured.

To summarize, Seybold and Hill postulated theoretically that psychological variables mediate the connection between spirituality and physical health [1]. At the same time, they mentioned a lack of knowledge about which psychological factors come into question to act as mediators. As there is empirical evidence that psychological resilience plays a significant role when people accomplish and maintain physical health, we assume that psychological resilience could be a mediator between different aspects of transcendental experiences and physical health. In the present study, the empirical focus is on the identification of spiritual predictors of psychological resilience. All in all, the present study addresses two research questions. The first aim is to investigate which aspects of spirituality may have a positive effect on psychological resilience. Specifically, three aspects of spirituality are measured: implicit self-regulation, mysticism, and meaning in life. Second, based on Büssing et al.'s findings [10, 11] age-related differences in the predictive power of the spiritual approaches will be investigated.

## 2. Materials and Methods

The study was conducted as a questionnaire survey. The questionnaires were mostly presented online; only a few were administered face-to-face. One part of the data collection was conducted as part of an summative evaluation at the end of an Austrian nationwide teacher training program. Second, people working at different hierarchical levels of an Austrian province government participated, mostly filling out the questionnaires in the paper-pencil version. Finally, staff and students of three Austrian universities were asked by email to participate in the online survey. Additionally, participants were asked to forward the link with the questionnaire to interested friends. Altogether, *N* = 265 people aged from 18 to 71 (mean = 33.4; Md = 29; SD = 12.2) participated, 192 (72.5%) females and 72 (27.2%) males. The sample was relatively highly educated: 133 (50.2%) had gained a higher-education entry qualification, and 119 (44.9%) had graduated from university, whereas only 11 (4.1%) had completed compulsory education and/or an apprenticeship and 4 (1.5%) had only completed compulsory education. Concerning employment status, 32 (12.1%) were university students (11 worked alongside their studies), 27 (10.2%) worked at the university, 43 (16.2%) were teachers, 54 (20.4%) worked in the civil service, and 70 (26.4%) were in private business. 

The questionnaire included three measures of spirituality, the Volitional Component Inventory (VCI), the Sources of Meaning and Meaningfulness Questionnaire (SoMe) and the Mystical Orientation Scale (MOS), and one outcome measure, the Resilience Scale (RS). All scales were constructed by calculating the mean of the particular items.

### 2.1. Volitional Component Inventory (VCI) [4]

The VCI (German version: [22]) is based on Fr$\ddot{\text{o}}$hlich and Kuhl's conception of self-regulation and measures different functional components that are differentiated into implicit and explicit self-regulation aspects. In this study, only implicit self-regulation is of interest, and the items are not original due to copyright issues, but they should help in having a better idea about the measured constructs. Implicit self-regulation includes the scales self-determination (Cronbach's *α* = .79; e.g., “almost everything I do, I do by choice.”), positive self-motivation (Cronbach's *α* = .81; e.g., “if I have to do some work, generally I can start with it immediately.”), self-calming (Cronbach's *α* = .83; e.g., “I can calm down when I feel effusively nervous.”), action oriented failure management (Cronbach's *α* = .84; e.g., “after a flop I can pick up courage very fast.”), and self-perception (Cronbach's *α* = .80; e.g., “under pressure, I do not lose the access to my feelings.”). All VCI scales consist of four items.

### 2.2. Sources of Meaning and Meaningfulness Questionnaire (SoMe) [28]

The SoMe, based on Schnell's theory of meaning in life, assesses 26 sources of meaning. It includes four dimensions (which are further divided into subscales): self-transcendence, self-actualization, order, and well-being and communality. In the present study, two scales were used that run across these dimensions: meaning in life (e.g., “I do have a life-task,” no original item) and crisis of meaning (e.g., “my life is useless,” no original item). Each of the two scales consists of five items and achieved good internal consistency: Cronbach's *α* was .79 for meaning in life and .91 for crisis of meaning.

### 2.3. Mystical Orientation Scale (MOS) [8, 25]

As described above, the MOS was translated from English to German by Schnell and Hanfstingl [26]. Empirically, three facets of mysticism were identified that are assumed to have differential relevance to laypeople [26]. The three scales had good internal consistencies in the present study: Cronbach's *α* was .90 for oneness and timelessness (e.g., “feeling myself at one with the universe,” no original item; the scale includes nine items), .83 for good power (e.g., “feeling moved by an ineffable force,” no original item; the scale includes five items), and .81 for spiritual insight (e.g., “having insights which I cannot put into words,” no original item; the scale includes seven items).

### 2.4. Resilience Scale (RS) [30]

The Resilience Scale was developed by Wagnild and Young [14], who proposed a two-dimensional approach to resilience, personal competence, and acceptance of the self. Wagnild and Young defined resilience as a personality factor that plays a moderating role for negative emotions and stress that helps to adapt flexibly to difficult situations. They validated their model of resilience in an empirical study [14]. Schumacher and colleagues [31] developed and validated a German version of the RS containing the two dimensions personal competence and acceptance of the self. In the present study, a new validated version of RS (RS-13) [30] was used and resilience was conceptualized one-dimensionally. The RS-13 consists of 13 items (e.g., “I have enough energy to do what I have to do,” no original item) and shows a very good internal consistency with Cronbach *α* = .93.

## 3. Results

### 3.1. Intercorrelations

Table 1 displays the bivariate correlations among the investigated variables. As expected, the two SoMe scales and all five VCI scales show moderate to strong correlations (|.42| to |.58|) to resilience. Quite unexpected, however, is the result that the three MOS have zero correlations to resilience. In order to exclude any nonlinear relationship between the MOS and resilience (e.g., a u-shaped link), the relational distribution plots of the two variables were examined, but no relevant nonlinearities were found.

However, all three MOS did show significant positive relationships among themselves and to the SoMe scale meaning in life. The fact that the in-between correlations of the MOS were not higher than about .65 justifies the separation into three individual dimensions. Furthermore, the three MOS showed significant negative (although low, <|.29|) correlations to action oriented failure management and self-perception. This supports the assumption of a highly differentiated dimensional structure of the measures of spirituality and transcendencies. 

### 3.2. Predicting Resilience from Measures of Spirituality

In order to investigate which dimensions of spirituality play a significant predictive role for psychological resilience, a multiple regression analysis was performed. A multiple regression design is able to identify collinearities; that is, two or more spirituality scales predict the same variance of psychological resilience. Additionally, variables which do not play a role in a bivariate correlational design may play a significant role within a pool of possible predictors. Thus, all spirituality variables were expected to predict resilience: meaning in life and crisis of meaning in the SoMe, self-determination, positive self-motivation, self-calming, action oriented failure management, self-perception of the VCI, and the three scales of the MOS, oneness and timelessness, good power, and spiritual insight.

In the whole sample, the ten predictors explained slightly below 50% of the variance in resilience (corrected *R* ^2^ = .47). As shown in Table 2, only three scales were significant predictors of resilience: crisis of meaning (negative predictor), self-determination, and positive self-motivation. As on the bivariate level (Table 1), the three MOS did not play a significant role for the target variable. In the following, the analysis was performed separately in two age groups. The young group aged from 18 to 29 (*N* = 137), and the older group (*N* = 124) aged from 29 to 71. The age of 30 was chosen as the split criterion for theoretical reasons. In many personality developmental theories the age of 30 is relevant for a life-span perspective on human development. From a sociocultural view, a prolonged adolescence should be completed finally at the age of 30. People from 30 onwards are often about to become parents themselves, their professional career courses are largely set, and their own parents may be growing old and may need more support. Personality psychology has found that personality is much more stable and different in quality from the age of 30 onwards than before (e.g., [32, 33]).

### 3.3. Predictors of Resilience: Young Group

Table 3 shows the results for the young group. The explained variance in the target variable is lower than that in the whole sample at about 40% (corrected *R* ^2^ = .42).

In the young group, only one variable is a significant predictor of psychological resilience: self-determination with a beta of .31. Self-determination is meant as a highly secular aspect of ego transcendence. All other spirituality scales do not have any predictive influence on psychological resilience.

### 3.4. Predictors of Resilience—Older People

In the older sample, several dimensions of spirituality predicted resilience. In the older sample, the ten variables explained 57% of the variance in resilience.

As Table 4 shows, positive self-motivation, self-perception, and the two MOS oneness and timelessness as well as spiritual insight were significant predictors of resilience. While the first three scales had a positive influence on psychological resilience, the MOS spiritual insight was a significant negative predictor (beta = −.25).

In fact, the relationship between the MOS and resilience becomes even stronger when the regression analysis is calculated only for participants aged 40 and older (*N* = 69). The negative path between spiritual insight and resilience grows to −.48**; oneness and timelessness (beta = .34*) and positive self-motivation (beta = .50**) are still positive predictors, whereas good power becomes insignificant (beta = .15).

## 4. Discussion

This study investigated the relationships of scales measuring different conceptions of spirituality to resilience in a sample of adults aged between 18 and 71. In the total sample, psychological resilience had significant connections to all meaning-of-life and internal-regulation scales but not to measures of mystical spirituality. Also, correlations revealed that one VCI scale action oriented failure management is also most of all negatively correlated with the MOS. Action oriented failure management focuses on action oriented (in contrast to state oriented) dealing with failure, whereas spiritual insight and also the other two MOS are characterized by holding and not acting. Insofar, the negative correlation between the MOS and action oriented failure management makes sense.

In a multiple regression analysis, only three variables remained significant predictors of psychological resilience: crisis of meaning, self-determination, and positive self-motivation. Meaning in life, self-calming, action oriented failure management, self-perception, and the three MOS do not show significant predictors. However, if the sample is divided into a younger and an older half at a cutoff age of 30 years, the predictors gain different relevance to psychological resilience. In the young sample only one rather secular predictor remains significant: self-determination. This result is consistent with the findings of Büssing [11]. In the older sample, the most powerful predictor is positive self-motivation, a measure of implicit self-regulation. In addition, two of the three MOS—oneness and good power—are significant positive predictors of resilience, while spiritual insight is a negative predictor. Moreover, taking people with age 40 and older as sample, the effect is even stronger. In this case, we probably have to speak about an age-related phenomenon.

A possible explanation for the result of negative prediction could be provided by psychological wisdom research and life-span developmental psychology [34]. In aging research it is a well-known phenomenon that an adaptive way of dealing with control and uncertainty is a relevant factor for well-being when people grow old. Here, being adaptive means that people have to deal with the fact that, on the one hand, many things in life stay uncontrollable and uncertain in the end, such as, for example, the loss of a job, prosperity, or an important friendship, or the death of close friends children, or spouses. For example, the Berlin wisdom paradigm [35] emphasizes the capability to recognize and manage uncertainty as one of the five criteria which are conditional for successful aging and wisdom. On the other hand, successful aging is closely linked to the acceptance of losing skills and competencies which are standard for younger people, for example, loss of physical healthiness or retardation of the ability for regeneration, (short-term) memory, concentration, or, in general, fluid intelligence. The dynamic between keeping or giving up control could be a possible explanation of the different relevancies of the MOS for psychological resilience. As mentioned above, spiritual insight is the only MOS scale that is characterized by keeping control when experiencing spirituality, whereas the other two MOS oneness and timelessness and good power address a positively connoted giving up of control. Perhaps striving for control in situations that cannot be controlled becomes an obstacle to resilience especially when people grow older or are faced with multiple stressors.

All in all, identifying the role of control and the loss of control in spiritual research could help concretize Seybold and Hill's [1] call for a better understanding of helpful and harmful effects of religion and spirituality on physical health via the pathway of psychological resilience.

## 5. Conclusions

This study presented empirical evidence that different aspects of spirituality play a role for psychological resilience in different life phases. In line with Büssing et al. [10, 11] for younger people secular aspects play a more strengthening role for psychological resilience than for older people. A surprising result is that, for people from the age of 30 and onwards, spiritual insight predicts psychological resilience negatively; the older people are, the stronger the effect becomes.

Following Kuhl [5] and the present results, it makes a difference for our psychological resilience whether we experience ego, self-, or spiritual transcendence. In this study, spiritual transcendence played a (positive or negative) role for psychological resilience only in people above the age of 29. Further research needs to investigate the extent to which cognitive development (e.g., increasing ambiguity tolerance) and the role of the experience of control are associated with the individual relevance of spirituality.

## 6. Limitations and Outlook

Due to the exploratory character of the research design, more research questions were raised than answered. For example, more precise theories that specify and test mediation models predicting health from spirituality with resilience as a mediator have to be developed and tested. Currently, we know that psychological resilience has a positive effect on physical health and that some dimensions of spirituality have this effect, too. The present study could not test such mediation models because physical health was not measured. In order to investigate the processes and mechanisms between spirituality, psychological resilience, and physical health more precisely, further research projects should consider longitudinal studies focusing on different dimensions of spirituality as well as spiritual development and physical health. The current results show us that there is variation in the influence of spirituality on psychological resilience by age. Therefore and against the background of the highly subjective and individual significance of spirituality, further research should include qualitative research exploring different qualities of transcendence. A further critical point of the study is that the respondents were highly educated and their health status was unknown. The results might be more differentiated with a database considering various health affections and different socioeconomic statuses.

## Figures and Tables

**Table 1:** Intercorrelations of the SoMe scale, VCI scale, and MOS with psychological resilience. *N* = 265.

	Resilience	SoMe		VCI					MOS	
		Meaning in life	Crisis of meaning	Self-determination	Pos. self-motivation	Self-calming	Action oriented failure management	Self-perception	Oneness and timelessness	Good power
SoMe										
Meaning in life	.44**									
Crisis of meaning	−.50**	−.57**								
VCI										
Self-determination	.54**	.41**	−.47**							
Pos. self-motivation	.58**	.37**	−.39**	.53**			
Self-calming	.47**	.30**	−.35**	.47**	.63**			
Failure management	.42**	.19**	−.35**	.41**	.43**	.52**			
Self-perception	.45**	.23**	−.44**	.44**	.38**	.44**	.60**			
MOS										
Oneness and timelessness	.01	.32**	.00	.02	−.09	−.17**	−.19**	−.16*	
Good power	.00	.32**	.00	.02	.00	−.12	−.18**	−.18**	.67**	
Spiritual insight	.05	.33**	−.02	.01	.02	−.06	−.16**	−.13*	.63**	.64**

**Correlations are significant at the level of .01 (two-tailed).

*Correlations are significant at the level of .05 (two-tailed).

**Table 2:** Multiple regression analysis. Dependent variable: resilience. Whole sample, *N* = 265. Significant predictors are bold.

Coefficients					
Model	Nonstandardized coefficients		Standardized coefficients	*T*	Significance
	Regression-coefficient *B*	Standard error	Beta		
Constant	2.368	.388		6.103	.000
Meaning in life	.112	.060	.120	1.865	.063
Crisis of meaning	**−.149**	**.063**	**−.148**	**−2.385**	**.018**
Self-determination	**.283**	**.099**	**.169**	**2.854**	**.005**
Pos. self-motivation	**.450**	**.093**	**.303**	**4.817**	**.000**
Self-calming	.047	.090	.033	.523	.601
Action oriented failure management	.076	.076	.061	.996	.320
Self-perception	.153	.078	.119	1.955	.052
Oneness and timelessness	.039	.060	.043	.643	.521
Good power	−.040	.062	−.042	−.639	.523
Spiritual insight	.033	.069	.030	.475	.635

**Table 3:** Multiple regression analysis. Dependent variable: resilience. Young sample, *N* = 137. Significant predictors are bold.

Coefficients					
Model	Nonstandardized coefficients		Standardized coefficients	*T*	Significance
	Regression-coefficient *B*	Standard error	Beta		
Constant	2.256	.581		3.886	.000
Meaning in life	.084	.098	.083	.854	.395
Crisis of meaning	−.151	.087	−.160	−1.737	.085
Self-determination	**.517**	**.153**	**.313**	**3.370**	**.001**
Pos. self-motivation	.187	.141	.125	1.326	.187
Self-calming	.127	.135	.085	.940	.349
Action oriented failure management	.053	.111	.041	.480	.632
Self-perception	.118	.107	.097	1.102	.273
Oneness and timelessness	−.011	.069	−.011	−.133	.910
Good power	−.086	.097	−.091	−.877	.382
Spiritual insight	.147	.102	.125	1.445	.151

**Table 4:** Multiple regression analysis. Dependent variable: resilience. Older sample, *N* = 124. Significant predictors are bold.

Coefficients					
Model	Nonstandardized coefficients		Standardized coefficients	*T*	Significance
	Regression-coefficient *B*	Standard error	Beta		
Constant	2.318	.481		4.824	.000
Meaning in life	.111	.071	.129	1.564	.121
Crisis of meaning	−.154	.091	−.130	−1.706	.091
Self-determination	.016	.124	.009	.126	.900
Pos. self-motivation	**.889**	**.124**	**.577**	**7.195**	**.000**
Self-calming	−.065	.111	−.049	−.587	.558
Action oriented failure management	.015	.099	.013	.149	.882
Self-perception	**.293**	**.111**	**.210**	**2.627**	**.010**
Oneness and timelessness	**.169**	**.072**	**.210**	**2.338**	**.021**
Good power	.076	.076	.083	.993	.323
Spiritual insight	**−.240**	**.093**	**−.251**	**−2.587**	**.011**
